# Progress in Laryngology

**Published:** 1895-10-12

**Authors:** 


### PROGRESS IN LARYNGOLOGY.

**Chronic Tonsilitis of Coli Bacillary Origin.**—A species of organism that has frequently been discovered in company with others in various forms of angina, is now known to be causally related to a special form of chronic tonsilitis. This is the *bacterium coli*, and the case in support of this assertion has been worked out and described by Lermoyez, Helme, and Barbier in the "Ann. de Mal. de l'Oreille" xx., 1894.[4] The following were some of the principal clinical features. A brief period of acute tonsillar inflammation was succeeded by a chronic course, but the local subjective symptoms were slight in comparison with the deterioration of the general system, manifested chiefly by digestive disorders. A dull white, softish exudate covered the tonsillar crypts, the intervening surface being red and free. This membranous coating was resistant, but slightly adherent, and bleeding did not occur upon its removal. Nothing short of excision of the tonsils made any impression upon it. There were no glandular enlargements. A pure growth of bacterium coli was obtained from the surface, and the microbe was abundant in the tissue. It differed from the normal type in not giving the indol reaction and in causing a very slow fermentation of lactose. The exudate has not the horny or gritty character of leptothrix pharyngo-mycosis, but cultivation is the best mode of distinguishing it therefrom. The mode of access was probably by drinking water, as the organism was found in the water that had been drunk by the patient.

**A New Scissors for Excising the Tonsils.**—We are all familiar with a form of hypertrophied tonsil, which, although less conspicuous on first inspection of the throat than that which projects freely towards the middle line, is none the less liable to inflammatory attacks, and to give rise to disagreeable symptoms. It lies packed between the two pillars, and is often firmly adherent to them, whilst by its tendency to grow upwards in a vertical direction it interferes seriously with the proper action of the soft palate and pharyngeal walls. Owing to this form of its growth, it is next to impossible to engage the mass in the ring of the guillotine or tonsillotome. To meet this difficulty, Arthur Ames Bliss[5] has designed a scissors adapted from Teet's nasal cutting forceps. The special features of the instrument are its long powerful handles, the relatively short strong blades, and a socket into which the shank of the lower blade falls as the scissors close; this socket arrangement presses the blades together, and prevents them from being sprung apart when cutting through thickened tissue. The scissors are used in conjunction with Farnham's crocodile jaw forceps, which firmly grasp the tonsil tissue, and do not slip away or tear out, as the volsella so often does. Raising the parts with these forceps, the scissors are passed beneath, and pieces of varying size are trimmed away as desired. Local anæsthesia is sufficiently well afforded by a 5 per cent. solution of cocaine applied on cotton pledgets. When ether is required, Bliss causes this agent to be administered to the patient in a recumbent posture on the couch, after which he is raised carefully to a sitting position by a trained assistant, who maintains him in it by standing on the right leg, and placing the left one bent at the knee behind the patient, whose back is thus supported against the assistant's leg, abdomen, and chest. The gag can be managed, and the position of the patient's head varied, to suit the requirements of the operator with great facility by the assistant. The tonsil forceps should be held in the hand nearest to the side to be operated upon. Hæmorrhage of an alarming character has never yet occurred under their use, but after the first few incisions it soon becomes apparent if one of those rare cases of hæmophilia is being dealt with, in which case excision can be discarded for other methods.

**Lingual Tonsil.**—The morbid conditions to which this body is subject are well worthy of careful study. Kronenberg[6] states that the commonest affection is hyperplasia (which practically mean an overgrowth of the normal lymphoid tissue of the part). The following are some of the more important symptoms which he describes: globus hystericus, a feeling of foreign body in the throat; pain, increased secretion, dry spasmodic cough, disturbance of voice, spasm of the œsophagus (a form of tenesmus), and local hæmorrhage. The latter is said to be apart from dilatation of the veins, which, however, are an invariable accompaniment of the lymphoid hypertrophy, and have given rise to the designation "pharyngeal piles." The hæmorrhage is important as liable to be mistaken for true hæmoptysis. Both veins and growth may coexist without giving rise to symptoms, and should not be treated in the absence of the latter. In slight cases, astringent gargles of myrrh or rhatany, or painting with various strengths of Schech's iodine solutions

(iodine, iodide of potassium, glycerine and carbolic acid, or menthol) are indicated. More pronounced hypertrophies can be removed with the snare, tonsillotome, or galvano-cautery burner. Dilated veins must be destroyed with the galvano-cautery if they give rise to hæmorrhage, but they usually disappear with the removal of the lymphoid masses. Acute inflammation (tonsillitis prœ-epiglottica acuta), ulceration, pharyngo mycosis, rec, may attack the lingual just as the faucial tonsils, and may be primary or secondary to affections of the latter. A similar and analogous treatment is indicated.

**Primary Tuberculosis of the Pharynx.**—In connection with the description of a case, Porter[7] mentions his method of administering creosote internally, which, in this instance, aided by local sprays and careful dieting and hygiene, resulted in both local and general improvement of the patient's condition. Only the best beechwood creosote is prescribed, and that largely diluted. A solution is commenced with, consisting of one drop to a tablespoonful of water, say twelve drops to six ounces. This is well shaken up, and half an ounce taken every three hours. The next solution may contain eighteen drops, and is so increased that the patient takes eighty or a hundred drops in twenty-four hours. But moderate doses, 40-50 drops in that time, give the best results. Sufficient water to prevent burning must always be added.

[4] Amer. Med. and Surg. Bull., Jan. 15, 1895 [5] Therapeutic Gazette, Mar. 15, 1895. [6] B.M.J., June 1, 1895. [7] Amer. Med. and Surg. Bull., April 1, 1895.

